# The spectrum and prognosis of Sjögren's syndrome with membranous nephropathy

**DOI:** 10.1093/ckj/sfae384

**Published:** 2024-12-11

**Authors:** Dan-dan Qiu, Zhi Li, Jing-jing Wang, Du-qun Chen, Yuan-mao Tu, Shao-shan Liang, Feng Xu, Dan-dan Liang, Ti Zhang, Zhen Cheng

**Affiliations:** National Clinical Research Center for Kidney Diseases, Jinling Hospital, Nanjing, China; National Clinical Research Center for Kidney Diseases, Jinling Hospital, Nanjing, China; National Clinical Research Center for Kidney Diseases, Jinling Hospital, Nanjing, China; National Clinical Research Center for Kidney Diseases, Jinling Hospital, Nanjing, China; National Clinical Research Center for Kidney Diseases, Jinling Hospital, Nanjing, China; National Clinical Research Center for Kidney Diseases, Jinling Hospital, Nanjing, China; National Clinical Research Center for Kidney Diseases, Jinling Hospital, Nanjing, China; National Clinical Research Center for Kidney Diseases, Jinling Hospital, Nanjing, China; National Clinical Research Center for Kidney Diseases, Jinling Hospital, Nanjing, China; National Clinical Research Center for Kidney Diseases, Jinling Hospital, Nanjing, China

**Keywords:** membranous nephropathy, PLA2R, prognosis, Sjögren's syndrome, spectrum

## Abstract

**Background:**

This study aims to investigate the spectrum and prognosis of membranous nephropathy (MN) in patients with Sjögren's syndrome (SS).

**Methods:**

SS patients with biopsy-proven kidney involvement who were diagnosed at our center between April 2007 and February 2024 were retrospectively reviewed and analyzed.

**Results:**

A total of 290 SS patients with kidney involvement were enrolled. The frequency of MN increased from 16.28% during the 2007–2010 period to 44.05% during the 2021–2024 period. After 2016, MN became the most common renal pathologic type, surpassing tubulointerstitial nephritis. PLA2R antibody or antigen was detected in 74 SS-MN patients, in whom 37 (50%) showed a negative result. Within the PLA2R-negative group, five out of 15 showed positivity for EXT1/EXT2 antigen and one out of eight for THSD7A antigen. Sixty-one SS patients with MN were followed up for >6 months, and 44 (72.13%) of them achieved renal complete remission (CR). Compared with PLA2R-negative patients, PLA2R-positive patients spent a longer time to achieve CR (1.46 ± 1.16 vs. 0.74 ± 0.47 years, *P* = .015) and had a higher rate of progression to the renal endpoint (8/32 vs. 1/29, *P* = .028). After adjusting for age, proteinuria, and eGFR, Cox regression analysis showed that PLA2R positivity remained a risk factor for CR [HR = 0.511, 95% CI (0.262 to 0.998), *P* = .049].

**Conclusions:**

MN has become the predominant renal pathologic type in SS. PLA2R-positivity testing followed by EXT1/EXT2 and THSD7A testing is recommended for SS-MN patients. Although most patients can achieve renal CR, the prognosis is usually poor in PLA2R-positive SS-MN patients.

## Graphical Abstract

**Graphical Abstract:**
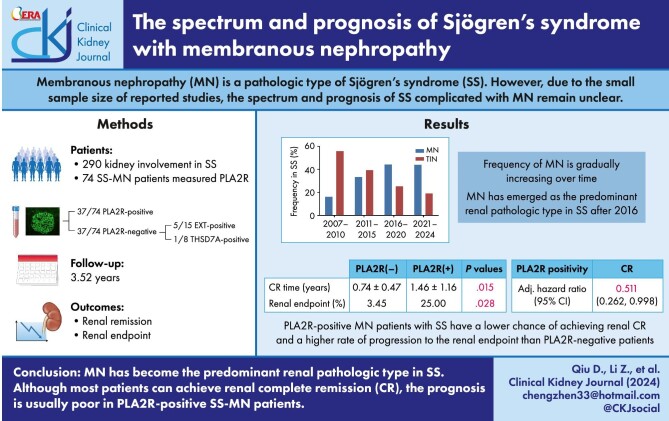

KEY LEARNING POINTS **What was known:**

- Sjögren's syndrome (SS) is a chronic autoimmune disease involving multiple organs including the kidney.
- Tubulointerstitial nephritis (TIN) was previously known as the most common renal pathologic type in SS with few studies reporting membranous nephropathy (MN).

**This study adds:**

- The frequency of MN is gradually increasing over time, and MN has emerged as the predominant renal pathologic type in SS.
- PLA2R-positivity testing followed by EXT1/EXT2 and THSD7A testing is recommended in SS patients.
- PLA2R-positive patients have a lower chance of achieving renal complete remission (CR) and a higher rate of progression to the renal endpoint than PLA2R-negative patients.

**Potential impact:**

- Our findings underscore the necessity of measuring serum PLA2R antibody and renal PLA2R antigen in SS-MN patients. More aggressive therapeutic strategies may be warranted for PLA2R-positive patients who fail to achieve renal remission.

## INTRODUCTION

Sjögren's syndrome (SS) is a chronic autoimmune disease typically presenting in females over the age of 50 years, with a female-to-male ratio of 9:1 [1]. SS is characterized by lymphocytic infiltration of the exocrine glands, especially the lacrimal and salivary glands, resulting in the well-known sicca symptoms of dry eyes and dry mouth [2]. Besides, SS can manifest systemic multi-organ involvement, which may include cutaneous vasculitis, interstitial lung disease, and kidney involvement [3].

Kidney involvement is an uncommon but a significant complication of SS, with a reported prevalence ranging from 1% to 33.5% [1, 4]. The clinical manifestations of kidney involvement in SS are variable, ranging from isolated electrolyte disturbances and nephrolithiasis to glomerulonephritis (GN) and tubulointerstitial nephritis (TIN) [1]. Kidney biopsy is the gold standard for diagnosing kidney involvement. Most studies indicate that TIN is the most common pathologic type in SS. On the other hand, GN is relatively rare, and includes membranous nephropathy (MN), membranoproliferative glomerulonephritis (MPGN), mesangial proliferative glomerulonephritis (MesGN), focal segmental glomerulosclerosis (FSGS), and other types [5].

Idiopathic MN is an autoimmune disease characterized by thickened glomerular capillary walls due to immune complex deposition and is the predominant cause of nephrotic syndrome in adults. The M-Type phospholipase A2 receptor (PLA2R) has been identified as the major podocyte antigen in 70%–80% of idiopathic MN. Anti-PLA2R antibodies are highly specific for the diagnosis of idiopathic MN [6]. However, recent studies have shown that serum antibodies and renal antigens of PLA2R may also be present in secondary membranous nephropathies, such as lupus nephritis, HBV-associated MN, cancer-associated MN, and SS [5, 7–9]. In addition, we find that the incidence of MN in SS is gradually increasing. However, due to the small sample size of reported studies, the spectrum and prognosis of SS complicated with MN remain unclear. To address this question, we conducted this retrospective study.

## MATERIALS AND METHODS

### Patients

**Figure 1:**
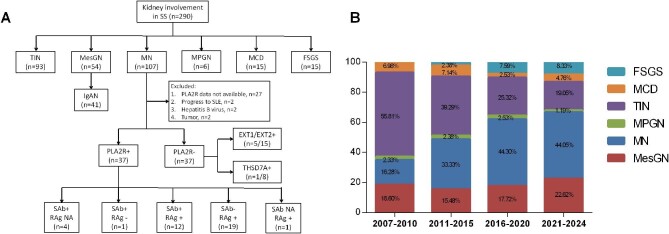
Prevalence and temporal trends in frequency of kidney involvement in SS patients. (**A**) The flowchart of patient inclusion and prevalence of different renal pathologic types in patients with SS. (**B**) Temporal trends in frequency of different renal pathologic types in SS patients. Abbreviations: MCD, minimal change disease; FSGS, focal segmental glomerulosclerosis; IgAN, IgA nephropathy; SLE, systemic lupus erythematosus; NA, not available.

We enrolled a total of 290 patients with biopsy-proven kidney involvement in Sjögren's syndrome (SS), diagnosed between April 2007 and February 2024 at the National Clinical Research Center for Kidney Diseases of Jinling Hospital (Nanjing, China). Inclusion criteria were as follows: (i) fulfillment of the 2016 ACR/EULAR classification criteria for primary SS [10]: patients tested positive for anti-SSA/Ro antibody and displayed focal lymphocytic sialadenitis with a focus score of ≥1 foci/4 mm^2^, each scoring 3; an abnormal Ocular Staining Score of ≥5 (or van Bijsterveld score of ≥4), a Schirmer's test result of ≤5 mm/5 min, and an unstimulated salivary flow rate of ≤0.1 ml/min, each with a score of 1; a total score of ≥4 meets the criteria for primary SS; (ii) biopsy-proven kidney involvement in SS; and (iii) exclusion of patients with other biopsy-proven concomitant kidney diseases such as Henoch–Schönlein purpura nephritis, diabetic nephropathy, and hypertensive nephropathy. Then, SS-MN patients were selected based on the following criteria: (i) biopsy-proven MN; (ii) detection of serum PLA2R antibody (SAb) or renal PLA2R antigen (RAg); and (iii) exclusion of viral hepatitis, tumor, or progression to systemic lupus erythematosus. The prevalence of different renal pathologic types in patients with SS is illustrated in Fig. 1A.

Among the SS-MN patients, those who were negative for both RAg and SAb were assigned as the PLA2R-negative group. Anyone deviating from this criterion was included in the PLA2R-positive group. Ultimately, 74 SS-MN patients were included in this study, and 37 patients were in the PLA2R-positive group (17 serum antibody-positive, 32 renal antigen-positive, and 4 without antigen detection). Available specimens obtained from PLA2R-negative patients were also stained for renal EXT1/EXT2, THSD7A, NELL1, SEMA3B, and PCDH7. The flowchart of inclusion in SS-MN patients is shown in Fig. 1A, and the details of SAb and RAg are available in Supplementary Appendix 1 This study was approved by the Ethics Committee of Jinling Hospital (2024DZKY-009–01) and was also in accordance with the Declaration of Helsinki. Written consent was not required for this noninvasive study, as determined by the ethics committee.

### Data collection and renal outcomes

Clinical and laboratory data of the patients within 1 month of kidney biopsy were collected as baseline data, including age, sex, 24-hour proteinuria, blood pressure, serum albumin, serum creatinine, estimated glomerular filtration rate (eGFR), serum immunoglobulin, serum PLA2R antibody, antinuclear antibody (ANA), anti-SSA antibody, anti-SSB antibody, anti-Ro-52 antibody, rheumatoid factor (RF), and serum complement factor 3 (C3) and C4. In addition, renal pathology, treatments, and follow-up records were also collected. The eGFR was estimated using the equation of the Chronic Kidney Disease Epidemiology Collaboration (CKD-EPI).

Complete remission (CR) was defined as <0.4 g/24 h proteinuria and stable renal function (±25%); partial remission (PR) as proteinuria levels <3.5 g/24 h and a >50% decline, along with stable renal function (±25%) no remission (NR); as failure to achieve CR or PR. Relapse was defined as an increase in proteinuria >1 g/24 h after a CR, or doubling of proteinuria after a PR [11, 12]. The renal endpoint was defined as a >30% decline in eGFR [13, 14] or end-stage kidney disease.

### Measurement of serum PLA2R antibody, renal PLA2R antigen, and other renal antigens

Serum PLA2R antibodies were measured by the indirect immunofluorescence assay (Euroimmune) before November 2015, and by the commercial ELISA assay (Euroimmune) afterwards. The results of PLA2R antibody <14 RU/ml by ELISA assay were considered to be negative, and ≥14 RU/ml were considered positive according to the manufacturer's protocol.

To detect renal PLA2R antigen, paraffin-embedded sections were deparaffinized, hydrated, and heated at 120°C for 10 minutes. The sections were then blocked with 10% fetal bovine serum for 10 minutes. The antigen was then conjugated with a rabbit polyclonal anti-human PLA2R antibody (Atlas Antibodies) followed by an FITC-conjugated swine anti-rabbit IgG antibody (Dako). The PLA2R antigen positivity was manifested as granular staining along the outer aspect of the glomerular capillary loop.

To detect renal EXT1/EXT2, NELL1, SEMA3B, PCDH7, and THSD7A antigens, paraffin-embedded sections were deparaffinized and rehydrated. After retrieval, the antigens were conjugated with anti-EXT1/EXT2, NELL1, SEMA3B, PCDH7 (Abcam Antibodies), and THSD7A (Sigma), followed by a horseradish peroxidase-conjugated secondary antibody. Staining was visualized using the DAB system.

### Pathologic study

All kidney biopsies were processed using standard techniques for light microscopy (LM), immunofluorescence (IF), and electron microscopy (EM) techniques. Kidney tubulointerstitial lesions were scored on a semiquantitative scale based on the percentage of the tubulointerstitial compartment affected in the cortex: 0, absent; 1, present in 1%–25% (mild); 2, present in 25%-50% (moderate); 3, present in >50% (severe). IF was performed on cryosections with fluorescein isothiocyanate-conjugated rabbit anti-human IgG, IgA, IgM, C3, C1q, and IgG subtypes, and κ and λ light chains (DAKO, Denmark). IF staining intensity was graded on a semiquantitative scale from 0 to 3+. Ultrastructural evaluation was performed with a Hitachi 7500 transmission electron microscope (Tokyo, Japan).

### Statistical analysis

Continuous variables are presented as the mean ± standard deviation (SD) for normal data, or as median and interquartile range (IQR) for skewed data. Categorical variables are expressed as proportions. Student's *t*-test or Mann–Whitney *U*-test was applied for continuous variables, and Chi-squared test or Fisher's exact test for categorical variables when comparing PLA2R-negative and PLA2R-positive groups. Kaplan–Meier survival analysis was performed to compare the CR rate between the PLA2R-negative and -positive groups. Cox regression analyses were used to identify independent risk factors for CR, and assumption of the Cox proportional hazards model was tested using Schoenfeld residuals. Results are expressed as hazard ratio (HR) with 95% confidence interval (CI). A *P* value <.05 was considered statistically significant. Data analysis was performed with SPSS version 21.0 software (SPSS, Armonk, NY, USA) and RStudio version 2023.03.0, an integrated development environment for R version 4.2.3 (R Core Team, Vienna, Austria).

## RESULTS

### Kidney biopsy findings in SS patients

A total of 290 patients with biopsy-proven kidney involvement in SS were enrolled in our center. According to the renal pathologic findings, GN was more common than TIN (67.93% vs. 32.07%). Patterns of GN lesions included MN (36.90%), MesGN (18.62%), minimal change disease (MCD, 5.17%), FSGS (5.17%), and MPGN (2.07%) (Fig. 1A).

After grouping into four periods from 2007 to 2024, there was a remarkable increasing trend in the frequency of MN, rising from 16.28% during the 2007–2010 period to 44.05% during the 2021–2024 period, while the frequency of TIN gradually decreased from 55.81% during the 2007–2010 period to 19.05% during the 2021–2024 period. The frequencies of MesGN and MPGN remained stable. After 2016, MN became the most frequent renal pathologic type, surpassing TIN in SS patients (Fig. 1B).

### Clinical characteristics of SS-MN patients

A total of 74 SS-MN patients were measured for serum PLA2R antibody or renal PLA2R antigen and included in this study. The mean age at the time of kidney biopsy was 48.58 years. Of them, 61 patients were female (82.43%) and 13 were male (17.07%), giving a female-to-male ratio of 4.69:1. Dry mouth was reported in 57 (77.03%) patients, dry eye in 44 (59.46%) patients, and decayed teeth in 24 (32.43%) patients. The most common immunoassay findings were positive ANA (95.95%) and anti-SSA antibody (95.95%), followed by anti-Ro-52 (86.49%) and anti-SSB (31.71%) antibodies. Notably, 31 (41.89%) patients presented with nephrotic-range proteinuria, and 60 (81.08%) had an eGFR >90 ml/min/1.73 m^2^. The median proteinuria was 2.90 (1.31, 4.63) g/24 h, and the mean serum albumin level was 30.22 ± 7.10 g/l. None of the patients presented with Fanconi syndrome.

According to serum PLA2R antibody and renal PLA2R antigen, the 74 SS-MN patients were classified into two groups: PLA2R positive group (*n* = 37, 50%) and PLA2R negative group (*n* = 37, 50%). The distribution of serum PLA2R antibody and renal PLA2R antigen is shown in Fig. 1A. Most patients in the PLA2R-positive group were positive for the glomerular PLA2R antigen. Among the PLA2R-negative patients, some patients were also stained for renal EXT1/EXT2, THSD7A, NELL1, SEMA3B, and PCDH7. Five out of 15 patients were positive for renal EXT1/EXT2 antigen, and one out of eight was positive for renal THSD7A antigen. Renal NELL1, SEMA3B, and PCDH7 were negative in all eight PLA2R-negative patients. The baseline hemoglobin level in PLA2R-positive group was lower than that in PLA2R-negative group (121.38 ± 15.48 vs. 129.78 ± 14.31 g/l, *P* = .018), and the serum C3 level was higher [0.94 (0.89, 1.08) vs 0.85 (0.75, 0.96), *P* = .003]. There were no significant differences in other clinical parameters, including age, sex, proteinuria, and eGFR between the two groups. The clinical characteristics of these SS-MN patients are shown in Table 1.

**Table 1:** Comparison of the characteristics of SS-MN patients between PLA2R-negative and -positive groups.

	All patients (*n* = 74)	PLA2R(−) (*n* = 37)	PLA2R(+) (*n* = 37)	*P* value
Age, years	48.58 ± 11.65	46.15 ± 11.26	51.02 ± 11.68	.072
Female, *n* (%)	61 (82.43%)	29 (78.38%)	32 (86.49%)	.359
Follow-up time, years	3.52 (1.22,5.41)	4.18 (1.24,5.32)	3.49 (1.22,5.45)	.583
Dry eyes, *n* (%)	44 (59.46%)	22 (59.46%)	22 (59.46%)	1.000
Dry mouth, *n* (%)	57 (77.03%)	26 (70.27%)	31 (83.78%)	.167
Decayed teeth, *n* (%)	24 (32.43%)	10 (27.03%)	14 (37.84%)	.321
Hypertension, *n* (%)	18 (24.32%)	8 (21.62%)	10 (27.03%)	.588
Diabetes, *n* (%)	5 (6.76%)	1 (2.70%)	4 (10.81%)	.358
Proteinuria, g/d	2.90 (1.31,4.63)	2.88 (1.28,4.40)	2.91 (1.28,4.69)	.935
Hemoglobin, g/l	125.58 ± 15.40	129.78 ± 14.31	121.38 ± 15.48	.018
Albumin, g/l	30.22 ± 7.10	30.28 ± 7.91	30.15 ± 6.29	.942
Globumin, g/l	24.70 (20.95,29.60)	26.70 (22.25,30.00)	22.45 (20.23,28.10)	.113
Scr, mg/dl	0.65 (0.55,0.75)	0.64 (0.55,0.76)	0.65 (0.56,0.75)	.940
eGFR, ml/min/1.73 m^2^	102.89 (92.30,113.05)	106.89 (95.37,113.51)	100.00 (90.80,112.85)	.517
IgG, g/l	10.35 (7.47,15.98)	10.30 (8.12,16.30)	10.40 (7.04,14.60)	.492
IgM, g/l	1.11 (0.73,1.72)	1.17 (0.76,1.80)	1.01 (0.71,1.52)	.290
IgA, g/l	2.80 (2.26,3.77)	2.86 (2.38,4.20)	2.72 (2.13,3.69)	.485
RF+, *n* (%)	20 (28.99%)	7 (20.59%)	13 (37.14%)	.130
Serum C3, g/l	0.91 (0.80,1.01)	0.85 (0.75,0.96)	0.94 (0.89,1.08)	.003
Serum C4, g/l	0.20 (0.16,0.26)	0.20 (0.11,0.25)	0.21 (0.18,0.27)	.098
SSA+, *n* (%)	71 (95.95%)	35 (94.59%)	36 (97.30%)	1.000
SSB+, *n* (%)	24 (32.43%)	12 (32.34%)	12 (32.34%)	1.000
Ro-52+, *n* (%)	64 (86.49%)	34 (91.89%)	30 (81.08%)	.174
ANA+, *n* (%)	71 (95.95%)	35 (94.59%)	36 (97.30%)	1.000

Abbreviation: Scr, serum creatinine. RF results were not available for five patients. Data are the mean ± SD, medians (25th and 75th percentiles) or proportions. *P* values were obtained from a Chi-squared test, Fisher's exact test, Mann–Whitney *U*-test, and Student's *t*-test as appropriate. A *P* value <.05 was considered significant.

### Pathologic findings in SS-MN patients

Kidney biopsies from all patients demonstrated typical pathologic features of MN, characterized by bright granular Ig staining along the capillary wall by IF, diffuse eosinophilic deposits, and electron dense deposits in the subepithelial position by LM and EM. There were 52 (70.27%) patients with global glomerulosclerosis, 16 (21.62%) patients with segmental glomerulosclerosis, and only one patient with cellular crescent. Most (82.43%) patients had mild interstitial fibrosis and tubular atrophy (IFTA). There was no significant difference in these features between the two groups. However, more patients in PLA2R-positive group developed mild and moderate acute TIN as compared with PLA2R-negative group (37.84% vs. 13.51%, *P* = .017). No patient developed severe acute TIN (Table 2).

**Table 2:** Comparison of kidney biopsy findings in SS-MN patients between PLA2R-negative and -positive groups.

	All patients (*n* = 74)	PLA2R(−) (*n* = 37)	PLA2R(+) (*n* = 37)	*P* value
Global glomerulosclerosis, *n* (%)	52 (70.27%)	26 (70.27%)	26 (70.27%)	1.000
Segmental glomerulosclerosis, *n* (%)	16 (21.62%)	7 (18.92%)	9 (24.32%)	.572
Crescent, *n* (%)	1 (1.35%)	0 (0%)	1 (2.70%)	1.000
Acute TIN, *n* (%)	19 (25.68%)	5 (13.51%)	14 (37.84%)	.017
0	55 (74.32%)	32 (86.49%)	23 (62.16%)	.017
1	17 (22.97%)	4 (10.81%)	13 (35.14%)	.013
2	2 (2.70%)	1 (2.70%)	1 (2.70%)	1.000
IFTA, *n* (%)	63 (85.13%)	32 (86.49%)	31 (83.78%)	.744
0	11 (14.86%)	5 (13.51%)	6 (16.22%)	.744
1	61 (82.43%)	30 (81.08)	31 (83.78%)	.760
2	2 (2.70%)	2 (5.41%)	0 (0%)	.493
IgG+, *n* (%)	73 (98.65%)	37 (100%)	36 (97.30%)	1.000
IgM+, *n* (%)	44 (59.46%)	20 (54.05%)	24 (64.86%)	.344
IgA+, *n* (%)	38 (51.35%)	20 (54.05%)	18 (48.65%)	.642
C3+, *n* (%)	73 (98.65%)	36 (97.30%)	37 (100%)	1.000
C1q+, *n* (%)	51 (68.92%)	26 (70.27%)	25 (67.57%)	.802
Brightest intensity for IgG, *n* (%)	72 (97.30%)	37 (100%)	35 (94.59%)	.493
IgG subtypes, *n* (%)	64 (86.49%)	33 (89.19%)	31 (83.78%)	.992
IgG1	62 (96.88%)	32 (96.97%)	30 (96.77%)	1.000
IgG2	56 (87.50%)	29 (87.88%)	27 (87.10%)	1.000
IgG3	53 (82.82%)	26 (78.79%)	27 (87.10%)	.379
IgG4	61 (95.31%)	31 (93.94%)	30 (96.77%)	1.000
Brightest intensity for IgG4, *n* (%)	45 (70.31%)	16 (48.48%)	29 (93.55%)	.000
IgG4+++, *n* (%)	14 (21.88%) 11	2 (6.06%)	12(38.71%)	.002

Kidney tubulointerstitial lesions used semiquantitative scores: 0, absent; 1, present in 1%–25%; 2, present in 25%–50%; 3, present in >50%. *P* values were obtained from a Chi-squared test and Fisher's exact test, as appropriate. A *P* value of <.05 was considered significant.

IgG, IgM, IgA, C3, and C1q staining was performed in all cases. IF analysis showed higher granular subepithelial deposits of IgG (98.65%) and C3 (98.65%) compared to IgM (59.46%), IgA (51.35%), and C1q (68.92%). IgG subtypes were stained in 33 PLA2R-negative patients and 31 PLA2R-positive patients (89.19% vs. 83.78%). All patients presented with polyclonal IgG subtype, and there was no significant difference in IgG subtype positivity between the two groups. IF staining intensity was graded on a semiquantitative scale from 0 to 3+. IgG4 staining had the brightest fluorescence intensity among the IgG subtypes in 16 patients of PLA2R-negative group and 29 patients of PLA2R-positive group (48.48% vs. 93.55%, *P* = .000). Notably, PLA2R-positive patients had a significantly higher rate of IgG4+++ than PLA2R-negative patients (38.71% vs. 6.06%, *P* = .002) (Table 2).

### Treatment and outcomes in SS-MN patients

**Figure 2:**
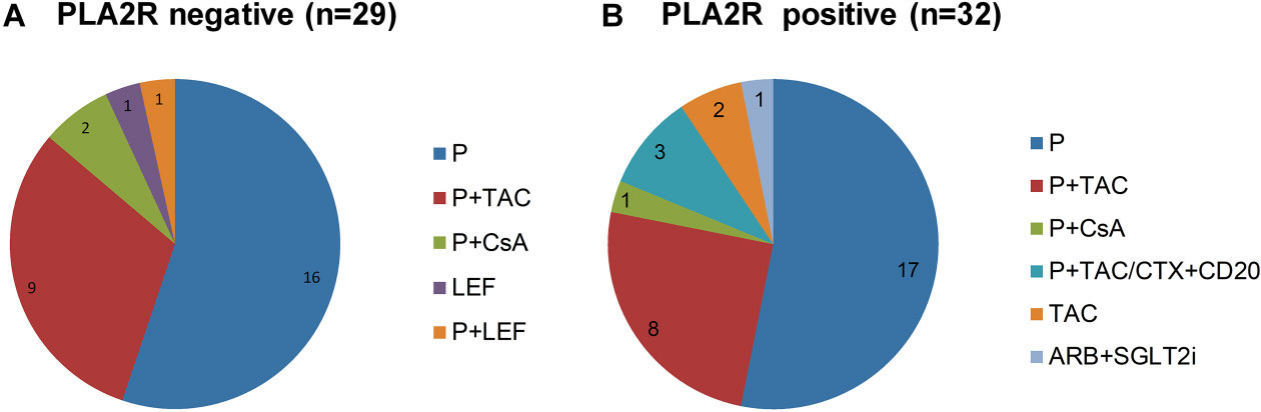
Treatments in SS-MN patients in PLA2R negative (**A**) and positive groups (**B**). Abbreviations: P, prednisone; TAC, tacrolimus; CsA, cyclosporine; LEF, leflunomide; CTX, cyclophosphamide; CD20, anti-CD20 monoclonal antibodies; ARB, angiotensin receptor blockers; SGLT2i, SGLT2 inhibitors.

Of the 61 patients (29 in PLA2R-negative group and 32 in PLA2R-positive group) who were followed up for >6 months, 24 (39.34%) patients were treated with corticosteroids in combination with other immunosuppressive agents, including tacrolimus (TAC) in 17 patients, cyclosporine A (CsA) in three patients, leflunomide (LEF) in one patient, and TAC or cyclophosphamide combined with anti-CD20 monoclonal antibodies in three patients. Other patients received monotherapy, including prednisone (P) in 33 patients, TAC in two patients, and LEF in one patient or allisartan combined with dapagliflozin in one patient for supportive treatment. The initial dose of corticosteroid, equivalent to prednisone, was 30 mg/d in most patients (range 10 to 30 mg/d). All patients received hydroxychloroquine and renin-angiotensin-aldosterone system inhibitors if tolerated. Treatment details between the two groups are shown in Fig. 2.

**Figure 3:**
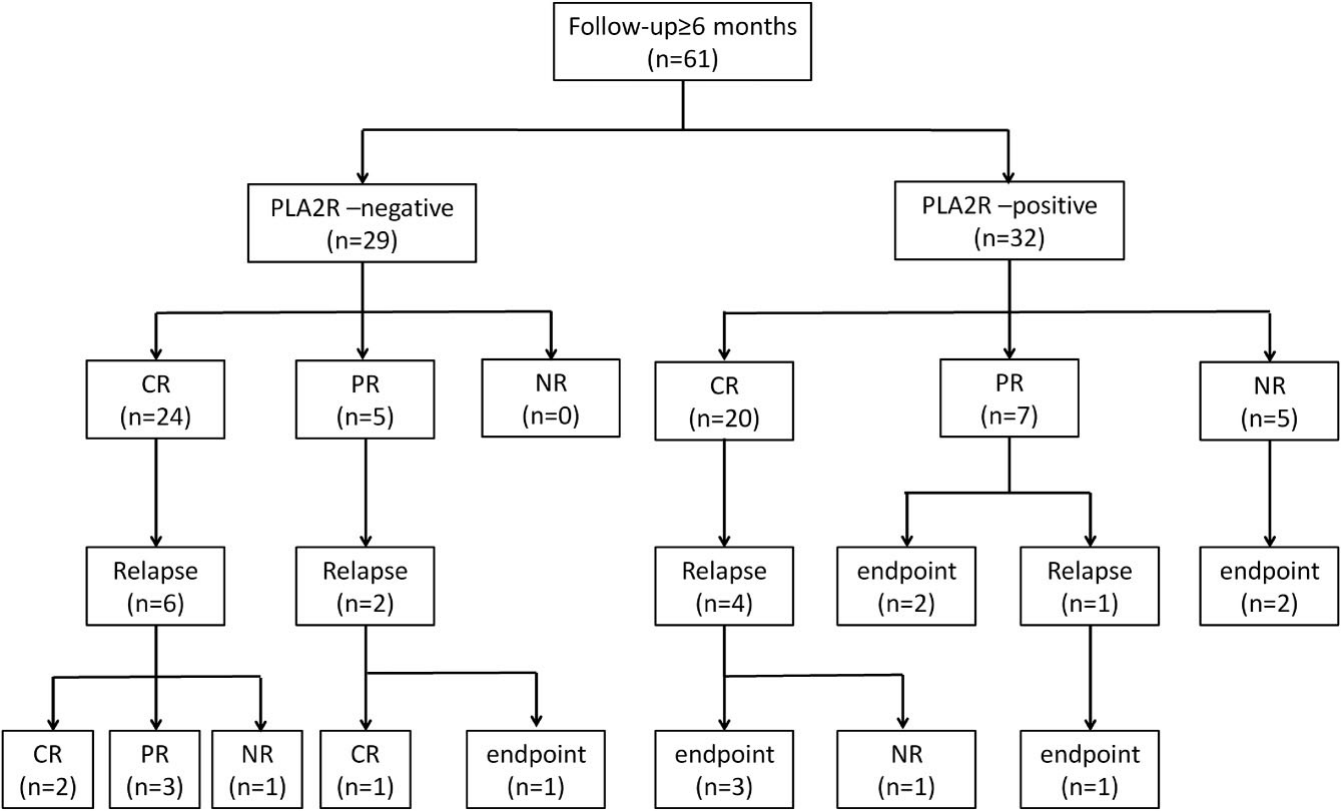
Renal outcomes in SS-MN patients in PLA2R-negative and -positive groups.

These 61 patients were followed up for 3.52 (1.22–5.41) years, during which 56 (91.80%) patients achieved renal remission, including 24 (82.76%) and five (17.24%) patients in the PLA2R-negative group who achieved renal CR and PR, respectively. By contrast, 20 (62.50%), seven (21.88%), and five (15.62%) patients in the PLA2R-positive group achieved CR, PR, and NR, respectively. Compared to the PLA2R-positive group, patients in the PLA2R-negative group had a significantly shorter CR time (0.74 ± 0.47 vs. 1.46 ± 1.16 years, *P* = .015). PLA2R-negative patients were more likely to achieve CR at 12 months (18/24 vs. 9/20, *P* = .042) (Table 3; Fig. 3).

**Table 3:** Comparison of the clinical outcomes in SS-MN patients between PLA2R-negative and -positive groups.

	PLA2R(−), *n* = 29	PLA2R(+), *n* = 32	*P* value
Follow-up time, years	4.18 (1.24,5.32)	3.49 (1.30,5.25)	.618
Remission time, years	0.62 (0.38,0.92)	0.82 (0.39,2.16)	.104
CR time, years	0.74 ± 0.47	1.46 ± 1.16	.015
CR, *n* (%)	24 (82.76%)	20 (62.50%)	.078
0–12 months	18 (62.07%)	9 (28.12%)	.042
>12 months	6 (20.69%)	11 (34.38%)	
PR, *n* (%)	5 (17.24%)	7 (21.88%)	.649
NR, *n* (%)	0 (0%)	5 (15.63%)	.054
relapse, *n* (%)	8 (27.59%)	5 (15.63%)	.255
Renal endpoint, *n* (%)	1 (3.45%)	8 (25.00%)	.028

Data are the mean ± SD, medians (25th and 75th percentiles) or proportions. *P* values were obtained from a Chi-squared test and Fisher's exact test, as appropriate. A *P* value of <.05 was considered significant.

In the PLA2R-negative group, eight (27.59%) patients entered renal remission but relapsed during follow-up, of whom six patients regained renal remission, one patient failed to achieve remission, and one patient reached the renal endpoint after treatment adjustment. By contrast, in the PLA2R-positive group, five out of 27 patients relapsed during remission, and four of them progressed to the renal endpoint. In addition, two patients with NR and two patients with PR in the PLA2R-positive group also culminated in the renal endpoint. Ultimately, eight (25.00%) PLA2R-positive patients and one (3.45%) PLA2R-negative patient progressed to the renal endpoint (*P* = .028) (Table 3; Fig. 3).

### Association of PLA2R with renal complete remission

**Figure 4:**
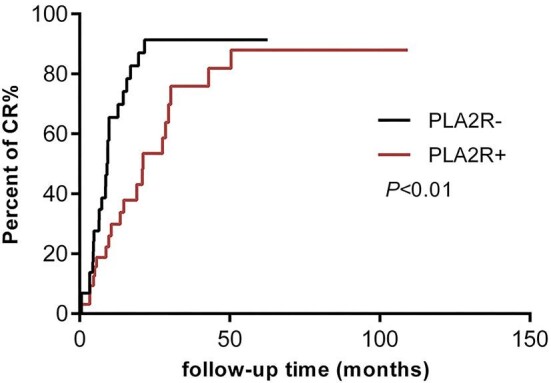
Kaplan–Meier analysis of renal CR in SS-MN patients in PLA2R-negative and -positive groups (*P* < .01).

In SS-MN patients, Kaplan–Meier analysis revealed that the PLA2R-negative group had a significantly higher rate of achieving renal CR compared to the PLA2R-positive group (*P* < .01). The cumulative renal CR rate after 12 months of treatment was 65.50% in PLA2R-negative patients and 29.88% in PLA2R-positive patients (Fig. 4). Univariate Cox regression analysis showed that PLA2R positivity was a risk factor for renal CR in SS-MN patients [HR = 0.452, 95%CI (0.247 to 0.829), *P* = .010]. After adjusting for baseline age, eGFR, 24-hour proteinuria, and acute TIN, PLA2R-positive patients still had a lower chance of achieving renal CR than PLA2R-negative patients [HR = 0.511, 95%CI (0.262 to 0.998), *P* = .049] (Table 4).

**Table 4:** Cox regression analysis of factors predicting renal CR in SS-MN patients.

	Univariate		Multivariate	
Variables	HR (95%CI)	*P* value	HR (95%CI)	*P* value
Age, years	0.962 (0.938,0.986)	.002	0.985 (0.956,1.014)	.249
Sex, male	1.299 (0.554,3.041)	.547		
24-h proteinuria, g/24 h	0.837 (0.739,0.949)	.005	0.855 (0.748,0.979)	.023
eGFR, ml/min/1.73 m^2^	1.025 (1.008,1.043)	.004	1.013 (0.997,1.030)	.109
Albumin, g/l	1.027 (0.981,1.075)	.254		
Hemoglobin, g/l	1.007 (0.988,1.027)	.480		
C3, g/l	0.296 (0.070,1.256)	.099		
Glomerulosclerosis	0.576 (0.305,1.088)	.089		
Segmental sclerosis	0.879 (0.443,1.744)	.713		
Acute TIN	2.436 (1.166,5.088)	.018	1.387 (0.607,3.170)	.438
PLA2R+	0.452 (0.247,0.829)	.010	0.511 (0.262,0.998)	.049

Data are the mean ± SD, medians (25th and 75th percentiles) or proportions. *P* values were obtained from a Chi-squared test, Fisher's exact test, Mann–Whitney *U*-test, and Student's *t*-test as appropriate. A *P* value of <.05 was considered significant.

## DISCUSSION

Altogether 290 patients were involved in this study, representing the largest cohort of biopsy-proven kidney involvement in SS study so far. The results of the present study suggest that MN has emerged as the most common renal pathologic pattern in SS patients. Most (72.13%) SS-MN patients in our study achieved renal CR. In addition, we demonstrated for the first time that 50% patients were PLA2R positive. Furthermore, five out of 15 (33.33%) patients were EXT1/EXT2 positive, and one out of eight (12.50%) was THSD7A positive. It was observed that PLA2R-positive patients required more time to achieve renal CR and were more likely to reach the renal endpoint.

Most studies suggest TIN as the most common renal manifestation that may develop in SS patients, with a prevalence ranging from 33% to 81% [1, 15], and MPGN as the most common and typical pattern of glomerular involvement, with a prevalence ranging from 8% to 30% [16–18]. In contrast to the global trend, an earlier study from China uniquely highlighted MN as the primary renal pathologic type in SS patients, with a prevalence of ∼50% [19]. It was found in our study that the frequency of MN tended to increase gradually over time and, after 2016, the incidence of MN surpassed that of TIN in SS patients. Similarly, a previous study from our center also showed that the frequency of idiopathic MN increased over time [20]. As the measurement of serum PLA2R antibody was not available in our center until 2013, it is possible that SS patients with lower proteinuria and normal renal function may not have undergone renal biopsies for MN detection, which could have resulted in a lower incidence of MN in SS before 2013.

Few studies have detected serum PLA2R antibody or renal antigen in SS-MN patients. Larsen *et al.* showed that one out of their six SS-MN patients had positive PLA2R staining [21]. One previous case report described a SS patient with chronic inflammatory demyelinating neuropathy who had MN lesions on kidney biopsy and was positive for PLA2R antibody [22]. In addition, a previous study in China showed that 8 out of 13 (61.54%) SS-MN patients were PLA2R positive [5]. Our study showed that 50% of the SS-MN patients were PLA2R positive. Previous studies have shown that PLA2R is a major target antigen in idiopathic MN and shows very low positivity in autoimmune-associated MN [7, 21]. It remains challenging to determine whether MN is a distinct disease that overlaps with SS or a unique pattern of SS-related kidney involvement in PLA2R-positive patients.

It was difficult to distinguish between the PLA2R-negative and -positive groups simply based on clinical and pathologic findings. However, despite similar treatment, PLA2R-positive patients spent a longer time achieving renal CR. Cox regression analysis showed that PLA2R positivity remained a risk factor for renal CR after adjusting for baseline age, eGFR, and 24-hour proteinuria. Similarly, a previous study showed that PLA2R antibody positivity was associated with poorer remission rates and prolonged remission times compared to negative patients with membranous lupus nephritis (MLN) [9]. In addition, we observed that PLA2R-positive patients were more likely to progress to the renal endpoint, especially those who relapsed or failed to achieve remission. These results demonstrate that it is necessary to measure serum PLA2R antibody and renal PLA2R antigen in SS-MN patients, and more aggressive therapeutic strategies may be warranted for PLA2R-positive patients who fail to achieve renal remission. Given the activation of B cells and production of autoantibodies in both SS and MN [23, 24], B cell-targeted therapies may provide new options.

In patients with negative PLA2R, EXT1/EXT2 staining was detected in both idiopathic MN and MLN, knowing that it was associated with autoimmune MN [25, 26]. In our study, we first demonstrated that five out of 15 (33.33%) SS patients with negative PLA2R were EXT1/EXT2 positive. In addition, one patient out of eight (12.50%) with negative PLA2R was THSD7A positive. These patients were carefully screened and followed up, and none was found to have a tumor, suggesting that EXT1/EXT2 and THSD7A may be associated with autoimmunity. Since several autoantigens have been identified in MN and MLN [27], it is possible that more antigens may be detected in SS-MN patients who were PLA2R negative. Given the evolving understanding of autoantigens in MN and MLN, an antigen-based classification of MN might be more appropriate than the traditional idiopathic versus secondary distinction [28].

Our study has several strengths. First, it enrolled the largest cohort of SS-MN patients in SS study so far. We also detected several autoantigens in these patients. Second, most of our patients were followed up for a sufficient time and ultimately achieved renal remission. Nevertheless, this study also had some limitations. First, as this is a retrospective study in a single center, whether our results can be generalized to larger populations requires further confirmation. Second, EXT1/EXT2 and THSD7A were only measured in a subset of PLA2R-negative patients. Thus, the true prevalence of EXT1/EXT2 and THSD7A positivity in SS-MN patients with negative PLA2R requires further clarification by more studies.

In conclusion, our findings reveal a significant increase in the frequency of MN in SS over time, with MN emerging as the predominant renal pathologic type. PLA2R detection, followed by EXT1/EXT2 and THSD7A testing, is recommended in these patients. Most patients can achieve renal CR and PLA2R positivity is associated with poor prognosis in SS-MN patients.

## Supplementary Material

sfae384_Supplemental_File

## Data Availability

The data underlying this article will be shared on reasonable request to the corresponding author.
